# Evaluation of Weight Loss Indicators and Laparoscopic One-Anastomosis Gastric Bypass Outcomes

**DOI:** 10.1038/s41598-018-20303-6

**Published:** 2018-01-31

**Authors:** Miguel A. Carbajo, Jose M. Jiménez, Enrique Luque-de-León, María-José Cao, María López, Sara García, María-José Castro

**Affiliations:** 1 Centre of Excellence for the Study and Treatment of Diabetes and Obesity, Valladolid, Spain; 2 Nursing Faculty, University of Valladolid, Valladolid, Spain; 3 Endocrinology and Clinical Nutrition Research Centre (ECNRC), University of Valladolid, Valladolid, Spain; 4 Castilla-León Regional Healthcare Management (Sacyl), Valladolid, Spain

## Abstract

Mini-gastric bypass**/**One-anastomosis gastric bypass (MGB-OAGB) is an effective bariatric technique for treating overweight and obesity, controlling and improving excess-weight-related comorbidities. Our study evaluated OAGB characteristics and resulting weight evolution, plus surgical success criteria based on various excess weight loss indicators. A prospective observational study of 100 patients undergoing OAGB performed by the same surgical team (two-year follow-up). Surgical characteristics were: surgery duration, associated complications, bowel loop length, hospital stay, and weight loss at 6 postoperative points. 100 patients were treated (71 women, 29 men); mean initial age was 42.61 years and mean BMI, 42.61 ± 6.66 kg/m^2^. Mean surgery duration was 97.84 ± 12.54 minutes; biliopancreatic loop length was 274.95 ± 23.69 cm. Average hospital stay was 24 hours in 98% of patients; no surgical complications arose. Weight decreased significantly during follow-up (*P* < 0.001). Greatest weight loss was observed at 12 months postsurgery (68.56 ± 13.10 kg). Relative weight loss showed significant positive correlation, with greatest weight loss at 12 months and %excess BMI loss > 50% achieved from the 3-month follow-up in 92.46% of patients. OAGB seems to be effective in treating obesity, with short hospital stays. Relative weight loss correlates optimally with absolute outcomes, but both measures should be used to evaluate surgical results.

## Introduction

Obesity has become the epidemic of this century, and is one of the biggest public health multifactorial problems due to genetic, social, or cultural factors^1^. According to World Health Organization global estimates, in 2014, 39% of adults were overweight and 13% were obese^2^.

Bariatric surgery has proven to be effective in weight loss and reduction of long-term associated comorbidities^3–5^. Among the bariatric mixed-type surgical procedures (restrictive and malabsorptive component), the laparoscopic one-anastomosis gastric bypass (OAGB), a modified mini-gastric bypass (MGB)^6^, characteristically offers effective, long-term weight loss results^7^.

At present, different indicators are used to express the results of postsurgery weight loss, such as percent excess weight loss (%EWL) and excess Body mass index (BMI) loss (%EBMIL). These have two different purposes: the first implies achieving a target weight listed as BMI of 25 kg/m^2^, while the other establishes surgical success or failure^8^. The method most widely used and accepted among surgeons is using the criteria initially described by Halverson and Koehler^9^ defining surgery success as %EWL > 50 and adding Reinhold’s^10^ result assessment based on final excess weight and ideal weight.

These indicators are different, causing the lack of consensus in defining target postsurgery weight, accepting BMI = 25 kg/m^2^ as successful treatment. Knowing initial weight and its postsurgery evolution is therefore necessary, not simply establishing surgery success using relative terms such as %EWL^11^.

The purpose of this study was to analyze weight evolution in patients from the first pre-surgery appointment through a 2-year follow-up, using different weight-reduction indicators and the involvement of other surgically-inherent determinants such as bowel loop length and surgery duration.

## Materials and Methods

A non-randomized prospective observational study of patients undergoing bariatric laparoscopic One-Anastomosis Gastric Bypass (OAGB) was performed at the Centre of Excellence for the Study and Treatment of Obesity and Diabetes in Valladolid (Spain). The University of Valladolid Institutional Review Board and its Ethics Committee for the Faculty of Nursing approved this study and the Helsinki-based experimental protocols prior to its undertaking.

From January 2010 through December 2010 (both months inclusive), 185 patients satisfying criteria for surgery were operated on using OAGB in the Centre of Excellence for the Study and Treatment of Obesity and Diabetes. Our study sample was composed of 100 patients with a 24-month postoperative follow-up.

The indication criteria for bariatric surgery accepted by the International Federation for the Surgery of Obesity (IFSO) are either BMI > 40 kg/m^2^, or BMI > 30–35 kg/m^2^ and presenting metabolic disease with inadequate monitoring or under medical treatment. All our study patients with an initial BMI > 30–35 kg/m^2^ had poorly controlled metabolic disease and were being treated by a specialist, meeting the criteria for surgical treatment.

Weight and BMI were determined at a pre-surgery appointment and subsequent postsurgery follow-up appointments at 3, 6, 9, 12, 18, and 24 months, according to accepted criteria to express weight loss^12^:

1 $${\rm{BMI}}=\mathrm{Weight}(\mathrm{kg})/{{\rm{height}}}^{2}({\rm{m}})$$

2 $$ \% {\rm{EBMIL}}=[({\rm{Preoperative}}\,{\rm{BMI}}-{\rm{current}}\,{\rm{BMI}})/({\rm{preoperative}}\,{\rm{BMI}}-25)]\times 100$$

3 $$ \% {\rm{EWL}}=[({\rm{Preoperative}}\,{\rm{weight}}-{\rm{current}}\,{\rm{weight}})/({\rm{preoperative}}\,{\rm{weight}}-{\rm{ideal}}\,{\rm{weight}})]\times 100$$

Ideal body weight was determined according to the Metropolitan Life Insurance Company formula. Due to excellent correlation between %EBMIL and %EWL, weight loss success can be categorized individually by %EBMIL, considering a result excellent if it exceeds 65%, good if between 50 and 65%, and a failure if under 50%^12,13^.

Other factors inherent to surgery were also studied, such as surgery duration, bowel loop length and perioperative complications.

As for postoperative complications, note that all patients operated in the Centre follow a postoperative protocol in which the medical and nutritional team participate, to improve technique efficacy and to induce improvements in the patients’ food behaviour, improving their postsurgical eating habits and preventing possible malnutrition deficiencies.

It is important to point out that if a patient needs pharmacological treatment, it is prescribed by the specialist regardless of the Centre’s postoperative protocol, which includes pharmacological supplements in the form of stomach protectors during the first month, calcium during the first 3 postsurgical months, and a multivitamin complex during the 12 postsurgical months.

Pre-surgery protocol included bariatric surgery selection criteria, psychological evaluation and standard analytical, radiological and cardiorespiratory function studies, with other complementary comorbidity studies^14^. Patients performed pre-surgery chest physiotherapy, physical exercise and active ambulation exercise, and followed a specifically designed pre-surgery dietary protocol preparation for 20 days. Complete liquid diet was then followed for eight days before surgery^15^.

### Surgical technique

**Figure 1:**
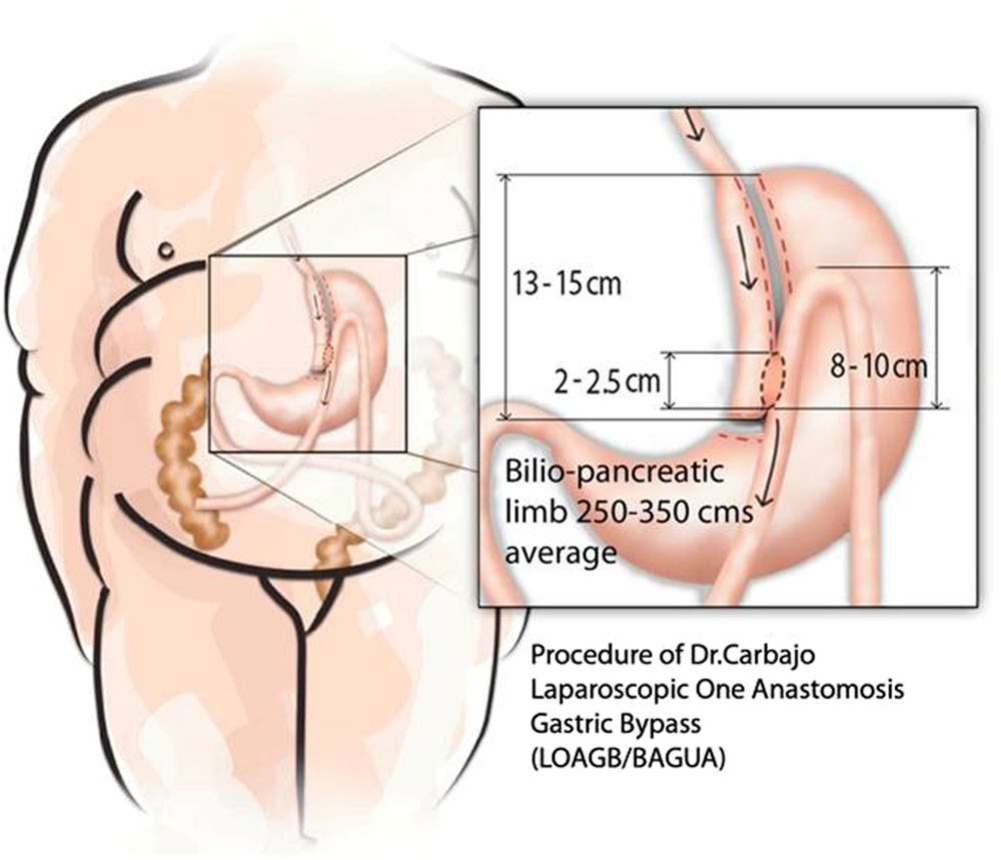
Graphic representation of the OAGB technique.

The OAGBs were performed using general anaesthesia and laparoscopic approach, as described previously^7,16^ (Fig. 1), by the same surgical team.

The entire small bowel was measured from the ligament of Treitz to the ileocecal valve, calculating the length of the common and the biliopancreatic limbs for each patient depending on total intestine length, patient age, sex, and race, the degree and model of obesity, BMI, associated comorbidities, metabolic syndrome, and even individual psychosocial factors.

### Statistical analysis

Data were analyzed using the statistical program SPSS version 15.0 (SPSS Inc., Chicago, IL). Quantitative variables were expressed as mean ± standard deviation (SD), and qualitative variables expressed using absolute and relative frequencies. The Chi-square test was used to study the association between qualitative variables. Student t test or Mann-Whitney test was used to study the differences between means for 2 groups, depending on the application conditions, and ANOVA or Kruskal-Wallis, for more than 2 groups. Statistical significance was set at *P* < 0.05.

## Results

A total of 100 patients (71 women, 29 men) were included in the study, with a mean age of 42.61 ± 11.33 years (range, 13–65 years). In the initial pre-surgery appointment, average weight was 116.75 ± 22.19 kg (range, 75–176 kg) and mean BMI was 42.61 ± 6.66 kg/m^2^ (range, 30–58.98 kg/m^2^); 65.51% of the male patients with obesity had a BMI > 40, while this figure was 53.52% for the females.

Twelve female patients (*P* = 0.017) received a cholecystectomy simultaneously with OAGB. Their mean age distribution was similar: 5 women < 42 years old and 7 women > 42.

Mean surgery duration was 97.84 ± 12.54 minutes, with no statistically significant difference between men and women (Table 1). Surgery for patients with higher initial BMI lasted longer. For those with type I obesity (BMI ≥ 30 ≤ 34.9 kg/m^2^), mean surgery duration was 98.4 minutes; patients with type II (BMI ≥ 35 ≤ 39.9 kg/m^2^) and type III (BMI ≥ 40 ≤ 49.9 kg/m^2^) had a mean of 96 minutes; and patients with type IV obesity (BMI ≥ 50 kg/m^2^) had a mean of 106 minutes (*P* < 0.05).

**Table 1:** General patient characteristics.

	Men (N = 29)	Women (N = 71)	*P* value
Age (years)	42.72 ± 12.25	42.56 ± 11.02	0.949
Height (m)	1.74 ± 0.09	1.61 ± 0.56	<0.001
Weight (kg)	132.23 ± 22.13	110.43 ± 18.99	<0.001
Body mass index (BMI) (kg/m^2^)	43.31 ± 6.49	42.32 ± 6.75	0.502
Surgery duration (minutes)	97.52 ± 11.30	97.97 ± 13.09	0.870
Biliopancreatic limb (cm)	289.31 ± 21.36	269.08 ± 22.15	<0.001

Another factor evaluated that increased surgery duration was combining cholecystectomy with OAGB in the same operation. Mean surgery duration of 121.58 ± 2.35 minutes was observed in patients undergoing both techniques simultaneously, and 94.60 ± 9.49 minutes with OAGB alone.

Mean biliopancreatic limb length was 274.95 ± 23.69 cm (longer in men) (*P* < 0.001). Correlating OAGB-derived bowel loop length and surgery duration revealed no statistically significant differences. Focusing on the 50th percentile (P50) of the bowel loops (270 cm in the biliopancreatic limb and 230 cm in the common loop), no statistically significant differences were found between surgery duration and biliopancreatic limb P50. However, comparing the common loop length P50 against duration, there was a difference: surgery involving a common loop > 230 cm took longer (101.87 minutes vs. 95.26 minutes) (*P* = 0.021).

Hospital stay for 98% of patients was 24 hours, and 48 hours for 2%. As for postoperative complications seen (Clavien-Dindo classification^17^), patients operated on under general anaesthesia fell into Grade III-b. During the first 30 postsurgical days, none of the 100 patients studied presented surgical complications, and all of them followed the Centre’s postoperative protocol under the control of the medical, surgical and nutritional team. After these 30 days, the controls continued for each of the review periods evaluated.

Weight loss is reported in absolute terms (kg) and relative terms: BMI, %EWL and %EBMIL (Table 2). Weight decreased significantly, from 116.75 ± 22.19 kg to 69.66 ± 13.07 kg, from the first postsurgery control up to 24 months monitored (*P* < 0.001). The greatest weight loss was observed at 12 months postsurgery (68.56 ± 13.10 kg). Although men weighed more in all controls, the reduction was significant in both sexes. Lowest mean weight was observed at 18 months for women (63.57 ± 10.96 kg) and at 12 months (78.96 ± 12.94 kg) (*P* < 0.001) for men.

**Table 2:** Post-OAGB weight loss evolution over 24 months, expressed in kg, BMI, %EWL and %EBMIL.

	Weight (kg)	BMI (kg/m^2^)	%EWL	%EBMIL
Pre-surgery	116.75 ± 22.19	42.61 ± 6.66^*^	—	—
3 months	81.55 ± 15.22^*^	29.77 ± 5.04^*^	66.86 ± 17.49^*^	78.72 ± 24.12^*^
6 months	74.02 ± 14.11^*^	26.99 ± 4.15^*^	81.05 ± 17.64^*^	95.50 ± 26.66^*^
9 months	72.13 ± 14.78^*^	26.44 ± 3.92^*^	83.31 ± 15.37^*^	96.96 ± 20.76^*^
12 months	68.56 ± 13.10^*^	25.08 ± 3.59^*^	89.70 ± 16.57^*^	104.82 ± 23.57^*^
18 months	69.67 ± 14.40^*^	25.27 ± 3.54^*^	88.40 ± 16.93^*^	103.43 ± 24.16^*^
24 months	69.66 ± 13.07^*^	25.33 ± 3.35^*^	88.10 ± 16.99^*^	103.79 ± 25.89^*^

The values are expressed as mean ± standard deviation.

*P < 0.001 compared with pre-surgical control. No patients (N = 100) were lost to follow-up.

BMI = body mass index; %EWL = percent excess weight loss; %EBMIL = percent excess BMI.

Pre-surgery BMI decreased significantly up to 24 months (42.61 ± 6.66 kg/m^2^ vs. 25.33 ± 3.35 kg/m^2^). At the last control evaluated, 48% of the patients had a normal weight (BMI ≥ 18.5 ≤ 24.9), and the cases of patients with morbid obesity (BMI ≥ 40) and super obesity (BMI ≥ 50) presurgery had been resolved.

**Figure 2:**
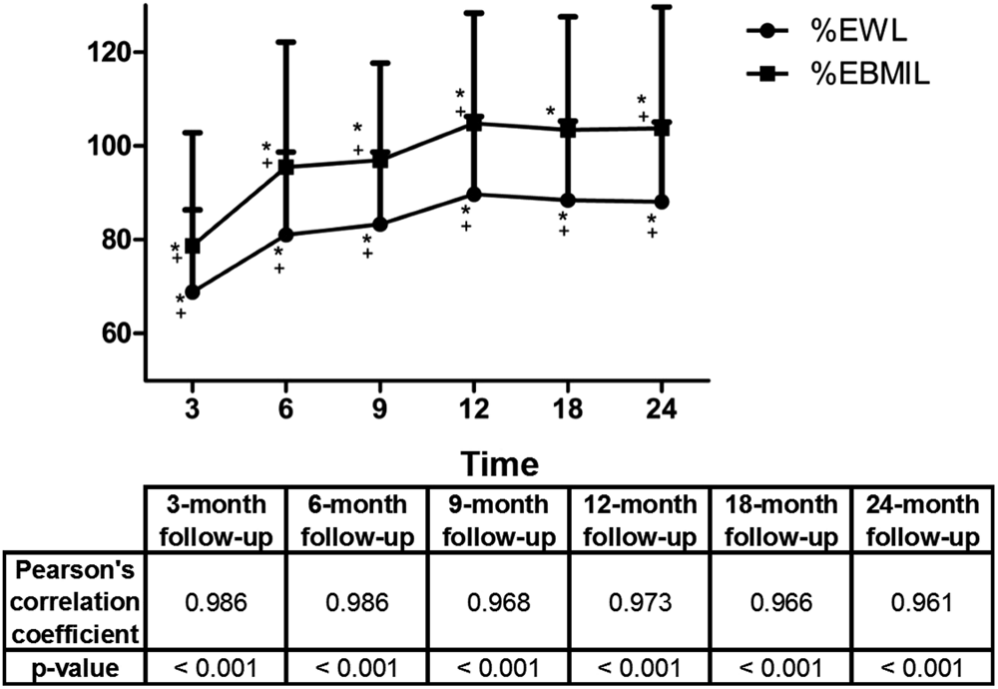
Postsurgery Evolution of %EWL and %EBMIL. Pearson’s correlation coefficient between %EWL and %EBMIL %EWL = percent excess weight loss; %EBMIL = percent excess body mass index. *P < 0.001 compared with the pre-surgery control. + *P* < 0.001 compared with the previous control. No patients were lost to follow-up.

Weight loss was also described in relative terms: %EWL and %EBMIL (Table 2). Both were significantly higher from postsurgery through the 24-month follow-up. The greatest weight loss was noted at 12 months postsurgery, as occurred with weight and BMI. From the first postsurgery control up to 24 months, %EWL rose from 66.86 ± 17.49% to 88.10 ± 16.99%, and %EBMIL changed from 78.72% ± 24.12% to 103.79 ± 25.89. At 24-months postsurgery, mean %EWL was higher for women than men: 91.34 ± 17.53% vs. 80.16 ± 12.64% (*P* < 0.001). Likewise, their 24-month %EBMIL was higher: women reached 107.98 ± 27.76% and men, 93.55 ± 17.04% (*P* < 0.05). The correlation coefficient between these two relative weight loss indicators (%EWL and %EBMIL) showed excellent correlation (Fig. 2).

**Figure 3:**
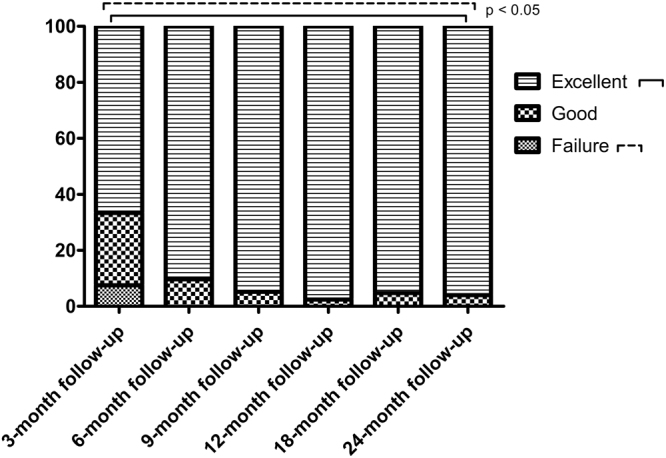
Result of bariatric surgery cataloguing based on postsurgery weight loss. Failure: %EBMIL < 50%; Good: %EBMIL = 50%-65%; Excellent: %EBMIL > 65%. No patients were lost to follow-up.

Weight loss success in this study was catalogued using Reinhold’s modified criteria^12,13^. An excellent result was considered %EBMIL > 65%; good, 50–65%; and failure, %EBMIL < 50% (Fig. 3). The number of patients with “failure” decreased significantly from the first postoperative evaluation (7.52%) to the 24-month follow- up; no patient showed “failure” in any other control. There were no statistically significant changes in patients with a “good” outcome at the initial control and at 24 months follow-up. However, patients with “excellent” criteria increased significantly by 2 years postsurgery (*P* < 0.001).

## Discussion

The use of OAGB technique has increased in recent years, obtaining positive results in long-term weight loss and being effective in controlling associated comorbidities and safe compared to other mixed surgery types^3,5,7^.

In our sample, females predominated (71%) and average age was 42.61 years. Our patient distribution by gender was similar to those in the studies by Wang *et al*. (79.43%), Piazza *et al*. (74.61%) and Noun *et al*. (66.1%), although their overall mean age was less than 41 years^18^.

Our OAGB duration was similar to other mini-gastric bypass techniques, although this clearly varies among different authors^18^: Rutledge *et al*.^19^ show shorter surgery time, 37.5 minutes on average. Hospital stay was 24 hours in 98% of our patients, unlike other studies, in which it ranges from 1 to 3.5 days^1,19^. Combining procedures in the same surgery increases its duration. Escalona *et al*.^20^ indicate a duration of 129.8 minutes when combining surgery with cholecystectomy, and hospital stay increases to 4 days. The patients in our series undergoing simultaneous cholecystectomy also had longer surgery time (121.58 minutes vs. 94.60 minutes).

Our mean biliopancreatic limb length (274.95 cm) was similar to that of other meta-analyses^18^ and longer than described in the work of Carbajo *et al*.^7^. Biliopancreatic limb length was significantly longer in our male patients, and they had higher mean initial BMIs than women. This is consistent with Noun *et al*.’s^21^ findings that the biliopancreatic or malabsorptive limb increases 10 cm for each pre-surgery BMI point > 40 kg/m^2^.

No surgical complications were observed in the patients studied. A very low percentage of complications is commonly found in this type of surgical techniques, with less than 6.7% of minor complications and less than 2% of major complications reported^18^.

Regarding weight reduction efficacy of mixed type bariatric procedures^18^, our patients’ mean initial weight (116.75 ± 22.19 kg) was slightly lower than in other studies such as Noun *et al*.^21^ (121.6 ± 23.8 kg) and Chakhtoura *et al*.^22^ (131 ± 23.1 kg). Our weight evolution followed the same trend as in those publications, observing the lowest mean weight at 12 months postsurgery (68.56 ± 13.10 kg), as in Chakhtoura *et al*.^22^ (89.8 ± 18.4 kg). In contrast, Noun *et al*.^21^ showed the lowest at 18 months postsurgery (79.3 ± 14.4 kg). Lee *et al*.^23^ compared weight loss efficacy of the MGB and Roux-en-Y gastric bypass (RYGB) using two homogeneously-aged patient subsamples. The initial weight of RYGB-treated patients was greater (119.1 ± 17 kg vs. 115.5 ± 17.5 kg); the lowest mean weight was reached at 2 years postsurgery, while other publications show the greatest weight loss at one year.

Our patients’ mean pre-surgery BMI was 42.61 ± 6.66 kg/m^2^. One year postsurgery, their mean BMI was 25.08 kg/m^2^, lower than the mean BMI (30.9 kg/m^2^) found in patients undergoing gastric sleeve procedure^24^. In our series, with OAGB, 48% of patients had normal weight (BMI > 18.5 < 25 kg/m^2^) at 24 months postsurgery.

In all studies describing relative weight loss, the correlation between %EWL and %EBMIL is excellent. Our results are 88.10% ± 16.99% and 103.79% ± 25.89%, respectively, at 2 years postsurgery. No authors using MGB^18,25^ show %EWLs as high as our 1-year postoperative values; Rutledge *et al*.^19^ is the closest (89%), followed by Carbajo *et al*.^7^ (75%) and Noun *et al*.^20^ (69.9%). Van De Laar *et al*.^11^ show a 2-year %EWL of 77.3% ± 22.8% in an RYGB patient series. Our study patients have a higher result (88.10% ± 16.99%).

%EBMIL is used to evaluate surgery success in terms of weight because it correlates excellently with %EWL, accepting BMI = 25 kg/m^2^ as normal weight and categorizing successful surgery as weight losses of %EBMIL > 50%^8–10^. In our series, 92.46% of patients undergoing OAGB achieve favourable results from the third postsurgery evaluation. From then up to 2 years, no patients show %EBMIL < 50%.

Many authors advocate the use of %EBMIL^26^, defining the objective normal weight previously, because the results of this indicator vary if a target normal weight is lower than the normal limit (BMI = 25 kg/m^2^)^8^. Weight loss outcomes should also be completed in relative terms using %TWL^11^.

One of the limitations of this study is the short-term follow-up of the sample selected; patient evolution should be completed with medium- and long-term data. Likewise, a possible bias to consider is that the sample of patients analyzed was not randomized. However, these limitations were acceptable to serve the purpose of this study.

This study, with a 2-year assessed follow-up, presents OAGB surgery as effective to treat obesity. Weight loss induced was greater than with other bariatric techniques. Using weight loss indicators is necessary for better surgery outcome standardization, although these indicators should be accompanied by absolute terms to assess surgery results correctly.

### Ethical approval

All procedures performed were in accordance with the ethical standards of the Valladolid University Institutional Review Board and its Ethics Committee for the Faculty of Nursing, and with the 1964 Helsinki declaration and its later amendments.

### Informed consent

Written informed consent was obtained from all study participants for their information to be stored in a database at the Centre of Excellence for the Study and Treatment of Obesity and Diabetes (Valladolid) and used for research.
