# Author Correction: Evaluation of Weight Loss Indicators and Laparoscopic One-Anastomosis Gastric Bypass Outcomes

**DOI:** 10.1038/s41598-018-24175-8

**Published:** 2018-04-26

**Authors:** Miguel A. Carbajo, Jose M. Jiménez, Enrique Luque-de-León, María-José Cao, María López, Sara García, María-José Castro

**Affiliations:** 1 Centre of Excellence for the Study and Treatment of Diabetes and Obesity, Valladolid, Spain; 2 Nursing Faculty, University of Valladolid, Valladolid, Spain; 3 Endocrinology and Clinical Nutrition Research Centre (ECNRC), University of Valladolid, Valladolid, Spain; 4 Castilla-León Regional Healthcare Management (Sacyl), Valladolid, Spain

Correction to: *Scientific Reports* 10.1038/s41598-018-20303-6, published online 31 January 2018

In the original version of this Article, María-José Cao was incorrectly affiliated with:

‘Centre of Excellence for the Study and Treatment of Diabetes and Obesity, Valladolid, Spain’

and

‘Nursing Faculty, University of Valladolid, Valladolid, Spain’

and

‘Endocrinology and Clinical Nutrition Research Centre (ECNRC), University of Valladolid, Valladolid, Spain’.

The correct affiliations are below:

‘Nursing Faculty, University of Valladolid, Valladolid, Spain’

and

‘Endocrinology and Clinical Nutrition Research Centre (ECNRC), University of Valladolid, Valladolid, Spain’.

